# Food-borne disease risk: biosurveillance in water networks

**DOI:** 10.2807/1560-7917.ES.2024.29.37.2400556

**Published:** 2024-09-12

**Authors:** Paulette Posen, Jane Heywood

**Affiliations:** 1 Centre for Environment, Fisheries and Aquaculture Science (Cefas), Weymouth, United Kingdom

**Keywords:** food-borne disease, biosurveillance, *Salmonella*, norovirus, *E. coli*

In 2018, food-borne disease (FBD) was estimated to be responsible for 2.4 million cases of illness and more than 16,000 hospitalisations per year in the United Kingdom (UK) [1], with revised estimates indicating ca 180 deaths annually in the UK arising from exposure to 11 key pathogens [2]. The estimated annual cost from these illnesses is EUR 10.5 billion (GBP 9 billion) [3], with far-reaching impacts on health providers, industry and individuals.

Food for human consumption can become contaminated at any stage of food production, delivery, storage or preparation, and can involve contamination from environmental, human or animal sources [4-6], making identification of sources and pathways of pathogens responsible for FBD outbreaks a complex process. Furthermore, there is currently no fully integrated framework in the UK for the monitoring and surveillance of FBD, causing difficulty in the prediction and delay in the mitigation of outbreaks.

In 2022, a series of programmes was launched under HM Treasury Shared Outcomes Fund, one of which, Pathogen Surveillance in Agriculture, Food and Environment (PATH-SAFE) [7], aimed to pilot a better national surveillance programme for FBD and antimicrobial resistance (AMR).

Pilot studies carried out under PATH-SAFE focused on norovirus, *Listeria monocytogenes* and *Salmonella* spp., identified by the Food Standards Agency (FSA) as being among the top five priority pathogens of concern with respect to FBD outbreaks [8], along with *Escherichia coli*, used as an indicator organism for faecal contamination.

To present the findings and discuss the challenges, needs and opportunities for implementing a successful national biosurveillance programme, the Centre for Environment, Food and Aquaculture Science (Cefas) and Bangor University hosted a workshop at the Royal Institution, London, on 31 January 2024. Stakeholders with a range of interests in pathogens implicated in FBD and AMR were invited to contribute their views and recommendations on how best to achieve the desired outcomes for an effective national surveillance framework. Participants from diverse roles (scientists, decisionmakers, public health analysts, policy advisors) across government, academia and charities attended the event.

The first part of the workshop was devoted to presentations of work carried out under the pilot studies, including a combined poster and networking session. For the second part of the workshop, participants were guided into breakout groups to participate in discussions on “Surveillance and management of microbiological risks: gaps & limitations, knowledge & perceptions, approaches & opportunities”.

This workshop was a collaboration across disciplines with participants from multiple organisations: Declan Power (Animal & Plant Health Agency); Jaime Martinez-Urtaza (Universitat Autonòma de Barcelona); Davey Jones, Kata Farkas, Reshma Silvester (Bangor University); Andrew Weightman (Cardiff University); Craig Baker-Austin, David Haverson, David Walker, Richard Heal (Centre for Environment, Fisheries & Aquaculture Science); Steve Morris (Department for Environment, Food & Rural Affairs); Edward Haynes (Fera Science Ltd); Anthony J. Wilson (Food Standards Agency); K. Marie McIntyre (Newcastle University); Mandy Wootton (NHS Wales); Ellie Brown (Ribble Rivers Trust); Oliver Pybus (Royal Veterinary College); Rob Collins (The Rivers Trust); Andrew Singer (UK Centre for Ecology & Hydrology); Matthew Wade (UK Health Security Agency); Edel Light (Veterinary Medicines Directorate).

## Presentations

After Edward Haynes (molecular biologist at Fera Science Ltd (Fera), and PATH-SAFE Science Fellow) gave a short informative presentation covering the context of the workshop and studies being carried out across the wider PATH-SAFE programme, the following presentations provided overviews of different elements of the pilot studies being conducted under PATH-SAFE.

### A multi-factor study of wastewater in the Taw & Torridge catchment

The presentation by David Walker (Environmental Microbiologist), Cefas, described one of the pilot studies, investigating prevalence and diversity of pathogens in weekly samples of raw influent and treated effluent at two sewage treatment works, over respective winter and summer sampling periods. The pathogens of interest (norovirus, *Listeria*, *Salmonella* and *E. coli*) were assessed against contemporaneous samples of river water and shellfish in the study area, to examine any spatial or temporal variability in their presence or persistence. The benefits of using wastewater surveillance as a preventative and mitigative measure for FBD outbreaks was highlighted.

### Assessment and prediction of the risks posed by hospital- and community-derived norovirus and AMR in coastal waters and shellfish

Kata Farkas (Environmental Virologist) and Reshma Silvester (Environmental Microbiologist), both Bangor University, presented a study examining the risk of viral release through effluent discharges from municipal and hospital wastes and, in particular, the potential for antibiotics in such discharges to contribute to the growing AMR problem. It was discussed how combining wastewater-based epidemiology with environmental surveillance can assist in identification of potential risks to human health from municipal and hospital discharges, helping to inform appropriate measures for minimising those risks.

### Food-borne pathogens affecting the Ribble shellfisheries: a review of available data and evidence

Ellie Brown (Strategic Evidence and GIS Manager), Ribble Rivers Trust, gave an overview of the characteristics of the River Ribble catchment, its natural environment, and human activities that may impact on the prevalence of food-borne pathogens in its estuarine waters, where three classified shellfisheries are located. The talk examined, among other things, land use, recreation, sewer misconnections, septic tanks, agriculture, livestock and high concentrations of both wild and farmed birds, all of which may act as sources or vectors of pathogen transmission in the catchment. Current monitoring and data availability were discussed, limitations highlighted and recommendations made for improved targeted surveillance in the catchment, including in partnership with ongoing programmes and initiatives.

### Understanding sources and pathways of estuarine pathogen loads: a tale of two models

Paulette Posen (Principal Investigator on PATH-SAFE Workstream 2a, presenting on behalf of Richard Heal (Spatial Environmental Scientist)); and David Haverson (Numerical Modeller), both Cefas, focussed on the terrestrial and aquatic aspects of modelling pathogen transport from river catchments to coastal waters, based on the Taw and Torridge pilot study carried out under PATH-SAFE. Firstly, the catchment model was considered, which included land use and wastewater discharge points as potential pathogen sources, using *E. coli* as the pathogen of interest to model likely transmission routes of faecal contamination from either humans or from agricultural livestock. The model considered how pathogen loadings and transport could be influenced by rainfall (hence river flow rates) and other environmental characteristics. The way in which model outputs could be calibrated and validated using existing monitoring data, or data collected during pilot study sampling, was conveyed. The second element of this presentation focused on the use of a hydrodynamic model to take outputs of *E. coli* loadings from the river catchment model, to assess pathogen transport and decay in estuarine and coastal waters. It was highlighted how model accuracy is constrained by availability of good quality data for model set up and validation.

### Machine learning assisted identification of pathogens in the microbiome of water systems

In the final presentation, Jaime Martinez-Urtaza (Molecular Epidemiologist), Universitat Autonòma de Barcelona (UAB), showed how novel machine learning techniques could be employed to build a picture of spatial and temporal pathogen prevalence and diversity from routine or study-specific sampling and analysis. By using whole genome sequencing techniques to generate new data from samples, and incorporating reference genomes from existing databases, these tools use decision-tree and optimisation methods to train the model to predict the presence or absence of pathogens of interest. As well as supporting the assessment of times and locations of highest risk with respect to pathogen contamination, the approach can be used to identify contamination events, and to achieve source tracking of implicated microorganisms with a very high resolution.

## Group discussions

Following the presentations, the workshop participants were split into breakout groups, ensuring a broad representation of occupations, disciplines and interests within every group.

Every group discussed the answers to four questions related to the establishment of an effective national biosurveillance framework for pathogens implicated in FBD, as follows:

1. What do you perceive are the gaps and limitations to the assessment and management of microbiological risk?
2. What do you see as the biggest opportunities for underpinning a future sustainable biosurveillance network?
3. What are the benefits or barriers to a ‘One Health’ approach to biosurveillance?
4. What do you see as the main benefits (and barriers) to emerging technologies/practices in the field of biosurveillance?

The activities prompted discussion on the perceived benefits from, and/or barriers to, improving and developing these systems and processes, including through new technologies. Data, collaboration and resources (both human and financial) were common themes throughout the discussions, with associated positive and negative aspects considered and identified across these themes. The aim was to draw out ideas for potential improvement and innovation, that could transform existing frameworks and translate into successful, cost-effective measures for a fully integrated national surveillance system for the prevention and mitigation of FBD outbreaks.

The issue of central leadership, highlighted under the ‘One Health’ discussion, was a cross-cutting topic of conversation, aligned with the need for joined-up priorities mapped across government, as raised in the wider discussions on gaps, limitations and opportunities. Within this mapping of objectives, there was also a need for forward thinking to anticipate the management of new and emerging risks. It was agreed that surveillance activities should be ‘co-productional’ from the outset, addressing multiple needs, using a common language and having a common strategic direction that could be used as levers for stakeholders.

It was generally agreed that there were both benefits and barriers to the mechanics and implementation of a holistic ‘co-productional’ approach to biosurveillance. For instance, implementation of parallel, multi-criteria assessments, via coordinated cross-organisational, multi-purpose monitoring, may lead to greater efficiencies or improved detection of combined pressures or previously unconsidered risks. However, correlating and interpreting the combined outputs across human, animal and environmental domains would be challenging, not least the digital processing power that would be required at the national level. Likewise, the holistic approach may be easier for some stakeholders to understand in the context of their real-world observations and experiences, but translation of findings from complex real-world interactions to an accessible language for a range of stakeholders can be difficult. The necessity to include uncertainty in analytical outputs was also stressed, even though many stakeholders still fail to take this into consideration. It was suggested that uncertainty could be described to stakeholders via a traffic light system, similar to that now used on some food products as an indicator of nutritional content (in which red, amber and green labels define whether a food has high, medium or low quantities, respectively, of fat, saturates, sugar and salt).

It was maintained that there needed to be an effective way of evaluating generalised models (e.g. meteorological/hydrological/hydrodynamic models for river catchment, estuarine and coastal transport modelling; geostatistical models for quantifying microbial risk; scenario modelling to evaluate future environmental risk) for their suitability to guide actions at local scales. One option would be to have a set of ‘characteristic’ models at regional catchment levels (taking account of, for instance, different natural geographical and human activity factors) which could then be further refined for application at the local level, taking specific features and characteristics (e.g. conurbations, land use, etc) into consideration.

One discussion group felt that timeliness was an issue in relation to the assessment and management of microbiological risk, both in terms of the timeliness in development and implementation of tools in various stakeholder strategies, and in the frequent disconnect between observations, reporting to regulators and mitigative actions. Timeliness was also seen to be an important consideration and/or constraint in emerging technologies and practices, which require a period of testing and validation before full implementation. It was thought that the costs and benefits of emerging technologies in biosurveillance were yet to be explored.

## Conclusions and recommendations

The workshop brought together opinions from different stakeholder perspectives on the gaps and limitations in current surveillance of pathogens in water networks, with a particular emphasis on FBD risk.

The main messages from the workshop regarding the establishment of a successful biosurveillance framework were:

- Collaboration across a wide range of stakeholders is vital.
- Availability, sharing and integration of high-quality data are crucial for risk assessment.
- Funding and human resources are important limitations to successful implementation.
- Citizen science and automated data collection are among the biggest opportunities for achieving this goal.
